# TBEV NS1 Induces Tissue-Specific Microvascular Endothelial Cell Permeability by Activating the TNF-α Signaling Pathway

**DOI:** 10.3390/ijms26115311

**Published:** 2025-05-31

**Authors:** Yana Khlusevich, Bogdana Kravchuk, Andrey Kechin, Alena Stepanova, Lyudmila Emelyanova, Sargis Khachatryan, Nina Tikunova, Andrey Matveev

**Affiliations:** 1 Institute of Chemical Biology and Fundamental Medicine, Siberian Branch of Russian Academy of Sciences, 630090 Novosibirsk, Russia; semali328@gmail.com (B.K.); a.a.kechin@gmail.com (A.K.); alena.o.lebedeva@gmail.com (A.S.); mila.kuharenko@mail.ru (L.E.); tikunova@niboch.nsc.ru (N.T.); 2 State Budgetary Healthcare Institution of the Novosibirsk Region, “State Novosibirsk Regional Clinical Hospital”, 630087 Novosibirsk, Russia; sargis-k@yandex.ru

**Keywords:** flavivirus, tick-borne encephalitis virus, NS1 protein, endothelial hyperpermeability, TNF-α signaling pathway, TEER

## Abstract

*Orthoflavivirus encephalitidis* (tick-borne encephalitis virus, TBEV) is of high concern due to its ability to cause severe neurological manifestations. Despite the fact that the role of NS1 proteins from various mosquito-borne flaviviruses in pathogenesis and their ability to affect human endothelial permeability have been investigated, TBEV NS1 has thus far been insufficiently studied. In this study, human endothelial permeability was assessed using TEER and transwell permeability assays. Signaling pathways were determined by RNAseq. The ability of the NS1 protein of TBEV to affect human endothelial permeability was investigated for the first time. It was shown that recombinant TBEV NS1 produced in eucaryotic cells directly affected human lung microvascular endothelial cells (HLMVECs) in vitro but not human umbilical vein endothelial cells (HUVECs). It was indicated that TBEV NS1 induced endothelial hyperpermeability of HLMVECs through activating TNF-α and other inflammatory signaling pathways.

## 1. Introduction

*Orthoflavivirus encephalitidis*, formerly known as tick-borne encephalitis virus (TBEV), is a flavivirus found in at least 27 countries in Europe and Asia. Tick-borne encephalitis (TBE) is diagnosed in 10,000–15,000 patients each year [1,2]. The infection is most often transmitted through the bite of an infected tick and, in rare cases, through the consumption of infected unpasteurized dairy products [3,4]. The virus can cause infections of varying severity, from asymptomatic infection to serious disease of the central nervous system, resulting in a variety of neurological symptoms and potential long-term outcomes, including death. The clinical manifestations of TBE consist of the first phase, characterized by headache and fever, and the second phase, in which myelitis can cause changes in consciousness, tremor, ataxia, and paresis [5]. Three recognized subtypes of the virus have been described, namely the European (TBEV-Eu), Siberian (TBEV-Sib), and Far Eastern (TBEV-FE) subtypes, as well as two recent Baikal (TBEV-Blk) and Himalayan (TBEV-Him) subtypes, which are characterized by different disease outcomes [1].

The TBEV genome is a positive-sense single-stranded RNA, approximately 11 kb in length [6]. The open reading frame is >10,000 bases long and is flanked by untranslated regions (UTRs). It encodes a polyprotein that undergoes co- and post-transcriptional processing and consists of three structural and seven non-structural proteins [7,8].

Non-structural protein 1 (NS1) of flaviviruses is a highly conserved protein of approximately 352 amino acid residues with a molecular mass of 46 to 55 kDa, depending on glycosylation [9]. Among the flaviviral non-structural proteins, NS1 is the only one secreted by infected cells [10]. NS1 glycosylation is important for its effective secretion and interaction with complements [11], and NS1 is a known inhibitor of complements and activator of toll-like receptors [12,13,14]. If NS1 is deglycosylated, the efficiency and replication rate of flaviviruses decrease [15]. NS1 can be found as a monomer (intracellular protein), dimer (membrane-bound protein), and hexamer (secreted protein) [16]. Intracellular NS1 plays a central role in flaviviral replication, while secreted and membrane-bound NS1 are involved in the anti-flavivirus immune response [17]. Flaviviral NS1 contains a glycosylphosphatidylinositol (GPI) anchor moiety, which facilitates the attachment of NS1 to the lipid rafts at the cell surface. GPI-anchored proteins are known to activate or inactivate intracellular signaling pathways when binding to specific antibodies or natural ligands [18].

The presence of the secreted form of NS1 (sNS1) has been reported in circulation during primary and secondary infections that elicit high concentrations of IgG. The anti-NS1 antibodies cross-react with a wide range of host proteins, namely human blood clotting factors, integrin/adhesion proteins, and components of the extracellular matrix [19]. It has been shown that monoclonal and polyclonal anti-NS1 antibodies can interact with fibronectin, plasma fibronectin, and peptides containing the RGD (arginine–glycine–aspartic acid) motif, which alters the normal functioning of the vascular system. This likely underlies the vascular leakage that occurs in patients with flavivirus infections [20]. It was found that deglycosylated NS1 mutant N207Q has a reduced ability to trigger endothelial layer glycocalyx (EGL) disruption and does not cause endothelial hyperpermeability in West Nile virus and Zika virus infections [12,18,19].

It has previously been shown that NS1 proteins of mosquito-borne flaviviruses selectively bind to a variety of human endothelial cells isolated from the brain, dermis, lung, liver, and ileal vein [21]. Treatment with NS1 proteins alters endothelial permeability in vitro and induces tissue-specific vascular leakage in mouse models of flavivirus infections, reflecting the pathophysiology of each flavivirus. Moreover, exposure to the NS1 from different mosquito-borne flaviviruses leads to differential disruption of endothelial glycocalyx components (EGL), resulting in endothelial hyperpermeability [21]. However, the molecular determinants of NS1, which are necessary to trigger EGL disruption, and the cellular pathways involved in this process, remain unknown [22]. Only the impact of the NS1 proteins of mosquito-borne flaviviruses on vascular permeability has been described [12,18,19,20,21], and no data on the impact of the TBEV NS1 protein on vascular permeability have been published.

In this study, the effect of the TBEV NS1 protein on vascular permeability was examined using human umbilical vein endothelial cells (HUVECs) and human lung microvascular endothelial cells (HLMVECs) as in vitro models of the vascular endothelium. The data obtained indicate that TBEV NS1 induces endothelial hyperpermeability in HLMVECs but not in HUVECs, and the activation of TNF-α and other inflammatory signaling pathways mediates the effects of the TBEV NS1 protein.

## 2. Results

### 2.1. Production and Purification of the TBEV NS1 Protein

The Sofjin strain belongs to the Far Eastern subtype, which is associated with a higher mortality rate [23]. To produce recombinant TBEV NS1 protein, the plasmid pSB-TBEV_NS1-sof was used. In this expression plasmid, the NS1 gene is located directly downstream of the leader sequence of the human albumin gene in the same open reading frame. The 6His-tag coding sequence required for Ni-NTA purification is located immediately after the NS1 gene. The plasmid pSB-TBEV_NS1-sof contains an ITR for the Sleeping Beauty transposase, the puromycin N-acetyl transferase gene, and the GFP gene (Figure S1).

**Figure 1:**
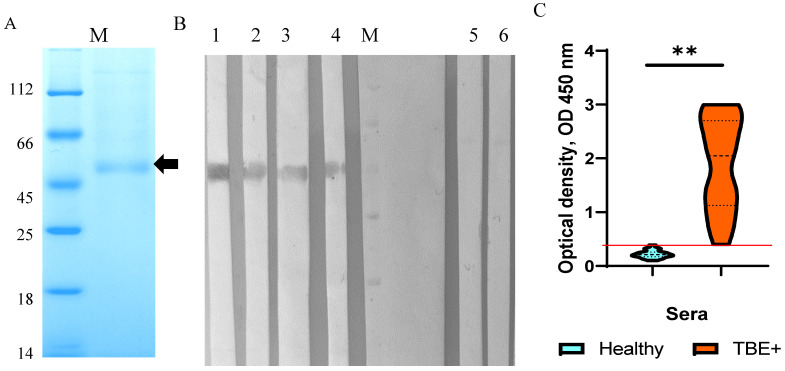
Characterization of recombinant TBEV NS1 protein purified from HEK293 cells. (**A**) SDS-PAGE of TBEV NS1. Band corresponding to recombinant NS1 protein is marked by black arrow. (**B**) Western blot analysis of the recombinant TBEV NS1 revealed by monoclonal antibodies against native TBEV NS1, namely NS1-1.3 (line 1), NS1-1.6 (line 2), NS1-2.299 (line 3), NS1-2.290 (line 4), and nonspecific monoclonal antibodies against TBEV glycoprotein E—14D5 (line 5) and FVN-32 (line 6); M—marker of molecular mass in kilodaltons. (**C**) ELISA of human sera from healthy donors (blue) and sera from patients with confirmed TBE (orange) for the ability to detect recombinant TBEV NS1. **—ρ < 0.05, (one-way ANOVA). Red line—cutoff level.

HEK293 cells were transfected with the constructed plasmid pSB-TBEV_NS1-sof. After selection with puromycin, a stable strain producing recombinant TBEV NS1 protein was obtained. The recombinant TBEV NS1 protein was purified from the culture medium by immobilized metal chelate affinity chromatography on Ni-NTA resin. Electrophoretic analysis of the purified recombinant TBEV NS1 protein indicated its homogeneity (Figure 1A). The electrophoretic mobility of the purified NS1 protein was consistent with its theoretically predicted molecular mass of 52 kDa. The purity of the purified TBEV NS1 protein, as estimated by PAGE, was approximately 80%. A total of 3.5 mg of purified NS1 protein was obtained from 1 L of culture medium. The purified TBEV NS1 protein was concentrated to a concentration of 1 mg/mL in PBS, filtered through a 0.22 syringe filter, and stored at 4 °C.

### 2.2. Characterization and Immunological Properties of the Recombinant TBEV NS1 Protein

The immunological properties and antigenic profile of the recombinant NS1 protein were evaluated by ELISA and Western blot analysis using the monoclonal antibodies NS1-1.3, NS1-1.6, NS1-2.299, NS1-2.290, and NS1-2.44. These monoclonal antibodies were previously obtained using hybridoma technology by fusing splenocytes from mice infected with a sublethal dose of the TBEV strain Sofjin with the murine myeloma cell line SP 2/0. Importantly, the monoclonal antibodies were selected using purified native TBEV NS1 protein [24]. Western blot analysis indicated that the monoclonal antibodies NS1-1.6, NS1-2.299, NS1-2.290, and NS1-2.44 revealed a band corresponding to the recombinant NS1 protein (Figure 1B). In ELISA, the above monoclonal antibodies bound to the HEK293-derived NS1 protein with the dissociation constants (Kd) similar to those of the native TBEV NS1 protein, whereas binding to the *Escherichia coli*-derived TBEV Trx-NS1 protein [24] was 2–10 times lower than that of the native protein (Table 1, Figure S2).

In addition, sera from patients with TBE and healthy donors [26,27] were used to test their binding to the HEK293-derived TBEV NS1 protein. The results indicate that the recombinant protein was detected by more than 95% of sera containing antibodies to the TBEV E protein, while the control sera from healthy donors did not react with the HEK293-derived NS1 protein NS1 (Figure 1C). Therefore, the obtained data confirm that the antigenic profile of the engineered recombinant TBEV NS1 protein corresponds to that of the native TBEV NS1 protein.

### 2.3. Effect of Recombinant TBEV NS1 Protein on Human Endothelial Permeability In Vitro

**Figure 2:**
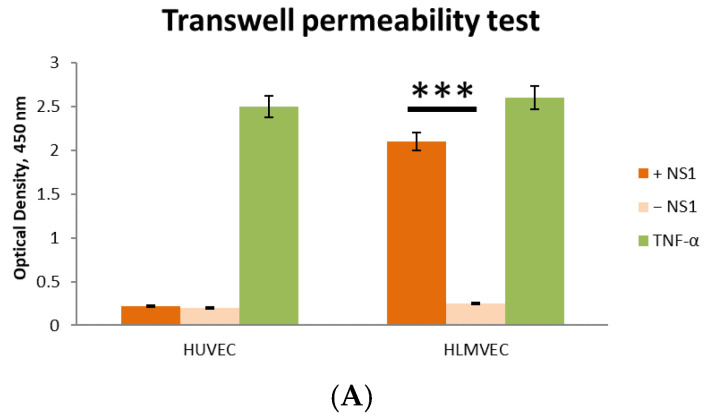

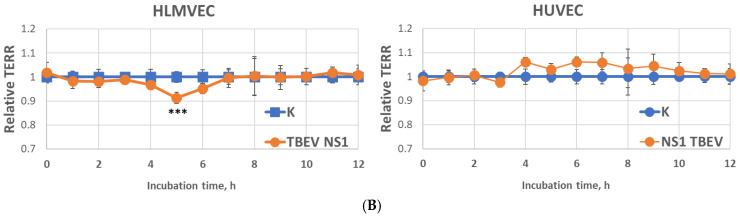
Evaluation of the effect of the TBEV NS1 protein on endothelial permeability at the indicated time points over 24 h. The permeability of human lung microvascular endothelial cells (HLMVECs) and human umbilical vein endothelial cells (HUVECs) was examined using (**A**) a transwell permeability test and (**B**) an EVOM3 Epithelial Volt/Ohm (TEER) real-time transendothelial electrical resistance assay. Results of the assay are shown in relative units. The cell index of control cells (not treated with TBEV NS1) was taken as 100%. Statistically significant differences between the TBEV NS1-treated group and TBEV NS1-non-treated group were evaluated by two-way ANOVA analysis using Dunnett’s test for multiple comparisons, with *** *p* < 0.01.

Two endothelial cell lines, HLMVEC and HUVEC, were used to study the effect of the TBEV NS1 protein on endothelial permeability. The results show that the TBEV NS1 protein had no effect on the permeability of the HUVEC endothelial monolayer in the solute flux assay (Figure 2A). In contrast, a 4-fold increase in endothelial monolayer permeability was observed for HLMVECs compared to the negative control during the first 5 min of the experiment.

The influence of TBEV NS1 protein on endothelial cell permeability was further verified by TEER measurements, and the obtained results confirm the data from the solute flux assay. For HLMVECs, an 8.7% decrease in monolayer resistance was observed compared to the control, which peaked after 5 h, indicating increased permeability of the human pulmonary capillary endothelial layer induced by the TBEV NS1 protein (Figure 2B). Monolayer resistance returned to its initial level 24 h after the start of the experiment. No effect of the TBEV NS1 protein on the permeability of the HUVEC line was observed (Figure 2A). The obtained results are consistent with previously reported data on the NS1 proteins of mosquito-borne flaviviruses. To determine the potential molecular mechanisms responsible for this process, RNA sequencing (RNA-seq) was performed.

### 2.4. Transcription Profile of Human Lung Capillary Endothelial Cells Treated with the TBEV NS1 Recombinant Protein

Given the specific effect of NS1 protein on the permeability of HLMVECs but not HUVECs, a transcriptional analysis of the former was performed using RNA-seq. Differential gene expression analysis was performed comparing the HLMVECs treated with TBEV NS1 protein and untreated control cells. The quality of the sequenced raw FASTQ files was assessed using FastQC software (version 0.11.9), and the data were then mapped to the Ensembl human genome reference version hg38 using STAR, resulting in read counts per gene and sample. Genes with a maximum read count of less than 10 in all samples were excluded from further analysis. Expression levels were compared by values obtained using Qualimap software (version 2.3). Normalization was performed by dividing the read count of each gene by the average read count for the respective sample and scaling by the highest average read count observed between the two samples. Differences in gene expression were calculated as the log2 ratio of read counts for each gene in the NS1-treated sample relative to the control (C) sample.

**Figure 3:**
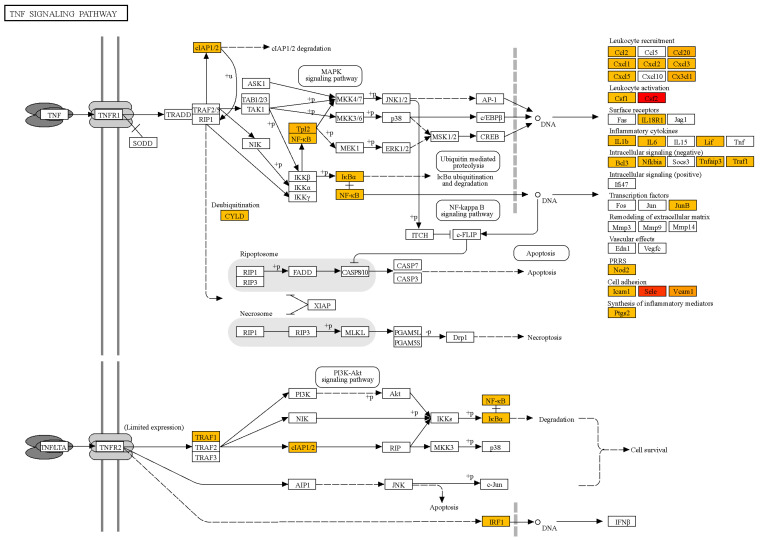
Scheme of the identified TNF signaling pathway, with genes identified by changes in mRNA levels in NS1-treated human lung microvascular endothelial cells (HLMVECs) compared to untreated control cells. Orange—mRNA level increased 2-fold, red—mRNA level increased 608-fold.

The analysis revealed that NS1 treatment significantly upregulated the mRNA levels of genes associated with endothelial activation, inflammation, and immune response in HLMVECs. Key genes with the highest fold changes included CSF2 (logFC = 9.2), SELE (logFC = 8.7), and VCAM1 (logFC = 6.9), which are involved in cytokine regulation and cell adhesion (Tables S1–S3). Gene ontology term enrichment analysis was performed using ShinyGO 0.75 for functional enrichment analysis to identify significantly overrepresented biological pathways. It was demonstrated that the genes associated with TNF-α signaling (TRAF1, TNFAIP2, and NFKBIZ) and oxidative stress regulation (SOD2) were significantly upregulated, suggesting that NS1 activates pathways critical for endothelial dysfunction and vascular inflammation (Figure 3). Chemokines such as CXCL1, CXCL8, and CCL2 were strongly upregulated, indicating a robust inflammatory response (Tables S1 and S2). These findings highlight the role of NS1 in promoting vascular inflammation through upregulation of cytokines and adhesion molecules.

## 3. Discussion

In most cases, TBEV infection occurs after a tick bite. The viral particles penetrate the body through saliva during tick feeding. The virus then multiplies locally in subcutaneous tissues, including the Langerhans cells (macrophages of the skin) and the neutrophils of the skin. Migrating monocytes/macrophages produce TBEV [28], and these cells can serve as vectors for transporting viral particles to lymph nodes. Replication of the virus in the lymph nodes leads to its dissemination into the bloodstream and the onset of viraemia. TBE is usually characterized by a biphasic course [1]. During primary viraemia, the virus affects various peripheral organs and tissues; however, infection often ceases at this stage, and seroconversion occurs without any obvious clinical signs [29]. The biphasic nature of TBE reflects the initial spread of the virus to peripheral tissues, which triggers a cytokine response, followed in some cases by penetration of the virus into the central nervous system (CNS), and a second neurological phase of the disease is recorded. It is known that the blood–brain barrier (BBB) consists of endothelial cells, astrocytes, pericytes, and adjacent neurons and plays a crucial role in brain homeostasis as well as in the formation of its microenvironment [30]. Virus penetration through the BBB is a prerequisite for CNS infection. This can lead to BBB dysfunction characterized by increased permeability, hypercellularity, and encephalopathy.

There are a number of various routes by which virus particles can cross the BBB: (i) induction of BBB permeability directly [31], (ii) infection of microvascular endothelial cells from the BBB front line [32,33,34], (iii) direct axonal retrograde transport from infected peripheral neurons spreading across neuromuscular junctions from muscles to somatic motor neurons in the spinal cord, and (iv) infection of olfactory neurons and spread to the olfactory bulb [35]. A so-called ‘Trojan horse’ mechanism has also been described for Zika and West Nile viruses, in which the virus is transported by infected immune cells that enter the CNS from the peripheral blood [36,37]. It has been shown that TBEV, Langat virus, West Nile virus, and Japanese encephalitis virus can enter the CNS without disrupting the BBB, and BBB permeability increases as a result of cytokine release in response to flavivirus replication in the brain [38,39,40,41]. Langat virus can probably also utilize an infection pathway via the olfactory nerve. After peripheral infection in mice, this virus was found first in the olfactory bulb and then spread to other brain regions [42]. It is still not entirely clear whether TBEV enters the brain via olfactory neurons or otherwise. It should be noted that most studies were performed in vitro or in animal models; however, such models may not adequately reflect the sequence of events occurring in the human body when TBEV penetrates the BBB.

Once TBEV enters the human brain, it multiplies in the large neurons of the anterior horns of the spinal cord, the medulla oblongata, the pontine, the dentate nucleus, the Purkinje cells, and the corpus striatum [1]. The main histological inflammatory responses observed after TBEV infection of the brain are lymphocytic-meningeal and perivascular infiltrates, as well as microglia proliferation with glial nodule formation and neuronophagia [1].

How TBEV crosses the BBB is not clear, and various mechanisms are probably involved in this process. One hypothesis is that TBEV neuroinvasion occurs by direct infection of microvascular endothelial cells. In an in vitro BBB model, it was shown that only 5% of microvascular endothelial cells were infected after the addition of TBEV; however, the infection persisted and spread through the BBB [43]. Notably, a primary brain vascular endothelial cell line was used in the study [43], which probably led to the presence of a subpopulation of neuronal cells that could be infected with TBEV.

Our data indicate that the NS1 protein of TBEV variously affects the permeability of vascular endothelial cells. Exposure of the endothelial cells of the small capillaries of the lung to this protein leads to an increase in their permeability, while it has no effect on the endothelial cells of the umbilical capillaries. The obtained data are consistent with studies on the effects of NS1 proteins of mosquito-borne flaviviruses, for which these proteins have been shown to differentially affect the permeability of different types of endothelium [21]. A disadvantage of this work is the lack of experiments with brain vascular endothelial cells. It was previously indicated that these cells are the most sensitive to treatment with different mosquito-borne flavivirus NS1 proteins, with the exception of NS1 YFV, which significantly reduces the permeability of the brain vascular endothelial monolayer [21].

Analysis of the enrichment of signaling and metabolic pathways with genes in the samples of NS1-treated cells compared to untreated samples shows the highest enrichment for the TNF signaling pathway (Table S3). Other signaling pathways detected are also associated with infectious and inflammatory processes. Thus, it is likely that treatment of endothelial cells with the TBEV NS1 protein triggers an inflammatory response in the endothelium, similar to the reaction induced by TNFa, which leads to disruption of the endothelial integrity through gap junctions between cells and thereby can contribute to the overcoming of the BBB by TBEV.

Previously performed RNA-seq of dendritic cells treated with TBEV NS1 [44] showed preferential downregulation of the genes encoding cytokines (TNF, Il1a, Il1b, Il6, Il12a, Il12b, Il15, Il27, and Il33) and chemokines (CCL5, CXCL5, CVCL9, CXCL10, CXCL11, and CXCL16), IFN-stimulated genes (ISGs, ISG15, and ISG20), and co-stimulatory molecules (CD40, CD80, CD83, and CD86) in treated cells compared to untreated cells. Our data indicate that endothelial cells treated with TBEV NS1 increase the expression of the genes involved in cytokine regulation and cell adhesion (SELE and VCAM1) and those encoding chemokines (CXCL1, CXCL8, and CCL2) and linked to TNF-α signaling (TRAF1, TNFAIP2, and NFKBIZ), as well as oxidative stress regulation (SOD2). This difference in results can be explained by the fact that when the NS1 protein appears in the bloodstream, TBEV can affect different types of cells in different ways. On the one hand, TBEV NS1 is able to suppress the proliferation and activation of T cells and inhibit the response to cytokine-mediated signaling in immune cells. On the other hand, it can activate inflammatory signaling and change cell adhesion when interacting with endothelial cells. Given the multidirectional effect of TBEV NS1 on different cells, the role of this protein in the pathogenesis of TBE has been underestimated, and the mechanisms of protein interaction with various tissues require further investigation.

## 4. Materials and Methods

### 4.1. Sera, Plasmids, and Bacterial and Mammalian Cells

Sera from patients with confirmed TBE (54% males and 46% females) hospitalized at Novosibirsk Infectious Disease Clinical Hospital No. 1 from April to September 2017–2019, and sera from healthy volunteers [25] were used in this study. Volunteers were healthy adults without chronic diseases (including autoimmune diseases) who had not been infected and/or hospitalized for at least six months. Informed consent was obtained from each patient and volunteer. The study was approved by the Ethical Committee of Novosibirsk Clinical Hospital of Infectious Diseases No. 1. Sera were stored at −70 °C after collection. All serum samples were tested for the presence of TBEV RNA using RT-PCR and for anti-TBEV protein E antibodies using a specific ELISA (both from Vector-Best, Novosibirsk, Russia).

Plasmids pET32a-TBEV_NS1-sof and pSB, *E. coli* XL1-Blue cells, and the HEK293 cell line were obtained from the Collection of Extremophile Microorganisms and Type Cultures of ICBFM SB RAS. Endothelial HLMVECs were kindly provided by Dr. Andrey Markov, ICBFM SB RAS. Endothelial HUVECs were kindly provided by Dr. Sargis Khachatryan, State Novosibirsk Regional Clinical Hospital. The TBEV-positive sera were obtained from the Collection of Extremophile Microorganisms and Type Cultures of ICBFM SB RAS.

HEK293 cells were cultured in a DMEM medium (Thermo Fisher Scientific, Waltham, MA, USA), supplemented with 2 mM GlutaMax I (Thermo Fisher Scientific, Waltham, MA, USA) and a 1-× antibiotic-antimycotic solution (Thermo Fisher Scientific, Waltham, MA, USA). The HLMVECs and HUVECs were cultured with EndoGRO-MV Complete Culture Media (Sigma Aldrich, St. Louis, MO, USA) in 24-well plates treated with rat-tail collagen, type I (Sigma Aldrich, St. Louis, MO, USA).

### 4.2. Construction of a Shuttle Plasmid Encoding the TBEV NS1 Protein

TBEV NS1 is known to be a highly conserved protein, with an identity between different TBEV subtypes of more than 95%. The gene encoding TBEV NS1 (GenBank accession number AEP20480) protein was amplified by PCR using the primers Start_NS1_TBEV_56_pSB 5′-GATGTTGGCTGTGCTGTGGACACTG-3′ and End_NS1_TBEV_56_pSB 5′-GTGATGGTGATGGTGATGTGCCACCACCATTGAGCGGACA-3′ and the pET32a-TBEV_NS1-sof as matrix [25]. The expression plasmid pSB and the PCR fragment were then digested with the restriction endonucleases EcoRV and BamHI (Sibenzyme, Novosibirsk, Russia) and combined in a ligation reaction. *E. coli* XL1-Blue cells (recA1, endA1, gyrA96, thi, and hsdR17(rK−, mK+), supE44, relA1, lac, [F′, proAB+, laclqZΔM15, and Tn10(Tetr)]) were transformed with the resulting ligation product, seeded on LB agar with ampicillin at a dose of 50 μg/mL, and cultured. Single colonies of *E. coli* cells containing plasmid pSB-TBEV_NS1-sof were screened by PCR using the same primers. PCR amplification conditions were as follows: 5 min at 95 °C; followed by 30 cycles of 30 s at 95 °C, 20 s at 56 °C, and 1.5 min at 72 °C; and a final extension of 6 min at 72 °C. The resulting PCR products were assessed by electrophoresis in a 1% agarose gel. The accuracy of the insertion of the gene encoding the TBEV NS1 protein was confirmed by Sanger sequencing using the primers E_SEQ_55U 5′-ACCAGAGTGATCGAGGCTGGGGG-3′ and E_SEQ_55L 5′-CAGCGACGTAATCCCCCGTATG-3′. The resulting plasmid was pSB-TBEV_NS1-sof, which encodes a TBEV NS1 protein with a His-tag at the C-terminus.

### 4.3. TBEV NS1 Protein Production and Purification

The adherent HEK293 cells were grown in a 6-well plate until they reached 70–80% confluence. Then the cells were co-transfected with the obtained plasmid, pSB-TBEV_NS1-sof, and pSB100x, which encodes the Sleeping Beauty transposase gene, using the PeiPRO transfection reagent (Polyplus, Strasbourg, France). The efficacy of transfection was assessed using flow cytometry and confocal microscopy, which evaluated the signal level of GFP in transfected cells 24 h after transfection. Cells were dissociated by trypsinization and resuspended in a phosphate buffer (PBS). Then, 48 h after transfection, the GFP-positive cells were sorted into 24-well plates containing selective media (IMDM, 10 FCS, 2 mM glutamate, 1× antibiotic-antimycotic solution, and 5 µg/mL puromycin) using a cell sorter SH800 (Sony Biotechnology Inc., San Jose, CA, USA). The selective medium was replaced with fresh medium every 3–4 days.

TBEV NS1 protein was purified from the culture medium by metal chelate chromatography on Ni-NTA agarose (Qiagen, Hilden, Germany) according to the manufacturer’s instructions. Purified TBEV NS1 protein was dialyzed to PBS and concentrated to a concentration of 1 mg/mL using Amicon centrifugal concentrators with a cutoff of 30 kDA. Purified TBEV NS1 was sterilized using a 0.22 μm syringe filter and stored at +4 °C.

### 4.4. ELISA and Western Blot Analysis

For indirect ELISA, 1 μg/mL of purified recombinant TBEV NS1 protein was added to each well of 96-well polystyrene plates (Greiner, Kremsmünster, Austria) and, after blocking the non-specific binding sites with a 5% skim milk solution, sera from TBE-positive patients at a dilution of 1:500 (N = 26) or monoclonal antibodies against TBEV NS1 protein (N = 5) at a concentration of 10 μg/mL were added [23,24]. After washing, the wells were incubated with rabbit anti-mouse IgG (Fc-specific) HRP-conjugated antibody (Biosan, Novosibirsk, Russia) or anti-human IgG (Fc-specific) HRP-conjugated monoclonal antibody X-53 (Biosan, Novosibirsk, Russia) for one hour at 37 °C. Immune complexes were visualized using tetramethylbenzi-dine-3,3,5,5 (TMB, Applichem, Darmstadt, Germany). Absorbance was measured at a wavelength of 450 nm using a microplate reader (Bio-Rad, Hercules, CA, USA). The affinity constant of mAbs was determined by ELISA, as previously described by Matveev et al. [26].

To determine the cutoff level for ELISA with sera from TBE patients, sera obtained from conditionally healthy donors who had not previously had TBE were used as a control. The mean optical density level of the control sera plus one standard deviation was taken as the cutoff. The ELISA signal levels of sera from TBE volunteers above the cutoff were considered positive.

The purified recombinant TBEV NS1 protein was separated using 12.5% PAGE and then transferred to a nitrocellulose membrane (Bio-Rad, Hercules, CA, USA). After blocking the nonspecific binding sites with a 3% bovine serum albumin solution (BSA, Amresco, Solon, OH, USA), the membrane was incubated with anti-TBEV NS1 protein monoclonal antibodies (N = 4) [24]. The membrane was then incubated with anti-mouse IgG (Fc-specific)–peroxidase antibody produced in rabbits (Biosan, Novosibirsk, Russia). Immune complexes were revealed using 4-chloro-1-naphthol (Applichem, Darmstadt, Germany). Immune ascitic fluid obtained from TBEV-infected mice was used as a positive control.

### 4.5. Solute Flux Assay

To evaluate the influence of the TBEV NS1 protein on the transit of macromolecules through the human epithelial cell monolayer, HLMVECs or HUVECs (60,000 or 80,000 cells per insert, respectively) were grown on collagen-coated PC membrane cell culture inserts for 24-well plates with a pore size of 0.4 μm and a diameter of 6.5 mm in a volume of 300 μL of EndoGRO-MV complete culture media per insert. Each insert was transferred into a well of a 24-well plate containing 1.2 mL of EndoGRO-MV complete culture media. One day before adding the TBEV NS1, 50% of the medium was replaced with fresh EndoGRO-MV complete culture media. Recombinant TBEV NS1 protein at a concentration of 10 μg/mL was added to an insert containing a monolayer of cells. Five hours after the start of the experiment, streptavidin conjugated with horseradish peroxidase (Sigma Aldrich) was added to the insert at a final concentration of 1 μg/mL and incubated for 20 min at 37 °C. Then, inserts were removed, and 100 μL of the culture fluid was collected from each well (lower chamber) of a 24-well plate. Horseradish peroxidase activity was determined using tetramethyl benzidine, and the concentration of the biotin-conjugated horseradish peroxidase from the insert into a well of a 24-well plate was determined by plotting a standard horseradish peroxidase curve. The signal was measured using an iMark plate reader (Bio-Rad). TNF-α (100 ng/mL) was used as a positive control, and untreated cell monolayers were used as a negative control.

### 4.6. Endothelial Permeability Test by Measuring Trans-Endothelial Electrical Resistance (TEER)

The permeability of endothelial cells treated with recombinant TBEV NS1 protein was assessed by measuring the TEER of these cells. A total of 50,000 HUVECs or HLMVECs were seeded in the PC membrane cell culture inserts for 24-well plates with a pore size of 0.4 μm and a diameter of 6.5 mm (Wuxi NEST Biotechnology Co., Ltd., Wuxi, China) in the volume of 300 μL of EndoGRO-MV complete culture media per insert. Each insert was transferred into a well of a 24-well plate containing 1.2 mL of EndoGRO-MV complete culture media. The 24-well plates with inserts were incubated at 37 °C and 5% CO_2_ until TEER ranges of 150–180 ohm (Ω) were reached. Recombinant TBEV NS1 protein at a final concentration of 10 μg/mL was then added to an insert containing a monolayer of cells. TEER values were measured at consecutive 1 h time points after the addition of TBEV NS1 using an EVOM3 epithelial volt/ohm (TEER) meter (World Precision Instruments, Sarasota, FL, USA) with “wand” electrodes (World Precision Instruments, Sarasota, FL, USA). Endothelial permeability was calculated as relative TEER using the following formula: (Ω treated endothelial cells − Ω medium)/(Ω untreated endothelial cells − Ω medium).

### 4.7. RNA-Seq

A total of 100,000 HLMVECs were seeded into the wells of a 12-well plate in a volume of 1 mL of EndoGRO-MV complete culture media. The 12-well plate was incubated at 37 °C and 5% CO_2_ until a monolayer of HLMVECs was formed. The cells were treated with 50 μL per well of recombinant TBEV NS1 protein at a concentration of 200 μg/mL in PBS or 50 μL of PBS alone and incubated at 37 °C and 5% CO_2_ for 3 h. The culture medium was then removed, and 1 mL of TRIzol™ reagent (Thermo Fisher Scientific, Waltham, MA, USA) was added to each well to lyse the cells. Cell lysates were then snap frozen and stored at −70 °C. Three biological replicates were included for each experimental condition to ensure statistical robustness. RNA isolation and RNA sequencing were performed in triplicate for both control and treatment groups at the laboratory of Genomed LLC (Moscow, Russia).

### 4.8. Statistics

Statistical analysis of ELISA results with TBEV-positive sera from patients with TBE and healthy volunteers was carried out using one-way ANOVA. Statistically significant differences between the TBEV NS1-treated group and the TBEV NS1-non-treated group were evaluated by two-way ANOVA analysis using Dunnett’s test for multiple comparisons. The Statistica 10 software package (StatSoft Inc., Tulsa, OK, USA) was used to perform statistical analysis.

## 5. Conclusions

In conclusion, the aim of this study was to comprehensively characterize the effects of TBEV NS1 on endothelial barrier function and the intracellular signaling cascades it triggers in order to better understand its contribution to TBEV pathogenesis and identify potential therapeutic targets.

## Figures and Tables

**Table 1:** Dissociation constants of monoclonal antibodies with native and recombinant TBEV NS1 protein measured by ELISA [25].

№	Monoclonal Antibodies	K_D_ Native TBEV NS1	K_D_ Recombinant TBEV NS1
1	NS1-1.3	8.51 ± 0.01 nM	8.21 ± 0.02 nM
2	NS1-2.299	0.20 ± 0.01 nM	0.22 ± 0.01 nM
3	NS1-2.290	0.35 ± 0.01 nM	0.36 ± 0.01 nM
4	NS1-2.44	0.68 ± 0.14 nM	0.67 ± 0.11 nM

## Data Availability

Data are contained within the article and Supplementary Materials.

## Supplementary Materials

The following supporting information can be downloaded at https://www.mdpi.com/article/10.3390/ijms26115311/s1.

## Abbreviations

The following abbreviations are used in this manuscript:

TBEV	Tick-borne encephalitis virus
TBE	Tick-borne encephalitis
NS1	Non-structural protein 1 of flaviviruses
EGL	Endothelial glycocalyx components
HUVECs	Human umbilical vein endothelial cells
HLMVECs	Human lung microvascular endothelial cells
sNS1	secreted form of NS1
GPI	Glycosyl-phosphatidylinositol
