# Supplementary material for: TBEV NS1 Induces Tissue-Specific Microvascular Endothelial Cell Permeability by Activating the TNF-α Signaling Pathway
Source: Int J Mol Sci. 2025 May 31;26(11):5311. doi: 10.3390/ijms26115311 (PMC12154905; doi:10.3390/ijms26115311)
Supplement: Supplementary file 1 [file ijms-26-05311-s001.zip › Supl figs.pdf]

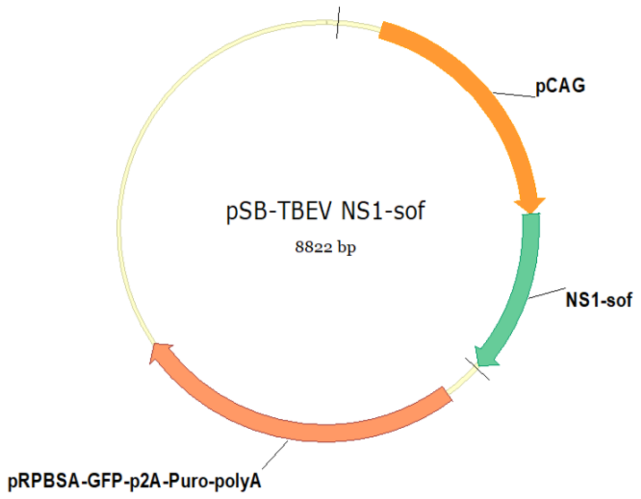

Figure S1. Scheme of plasmid map pSB-TBEV_NS1-sof. The pCAG promoter, TBEV sofjin NS1 protein genes, and puromycin resistance gene fused to GFP protein via p2a-peptide were labeled.

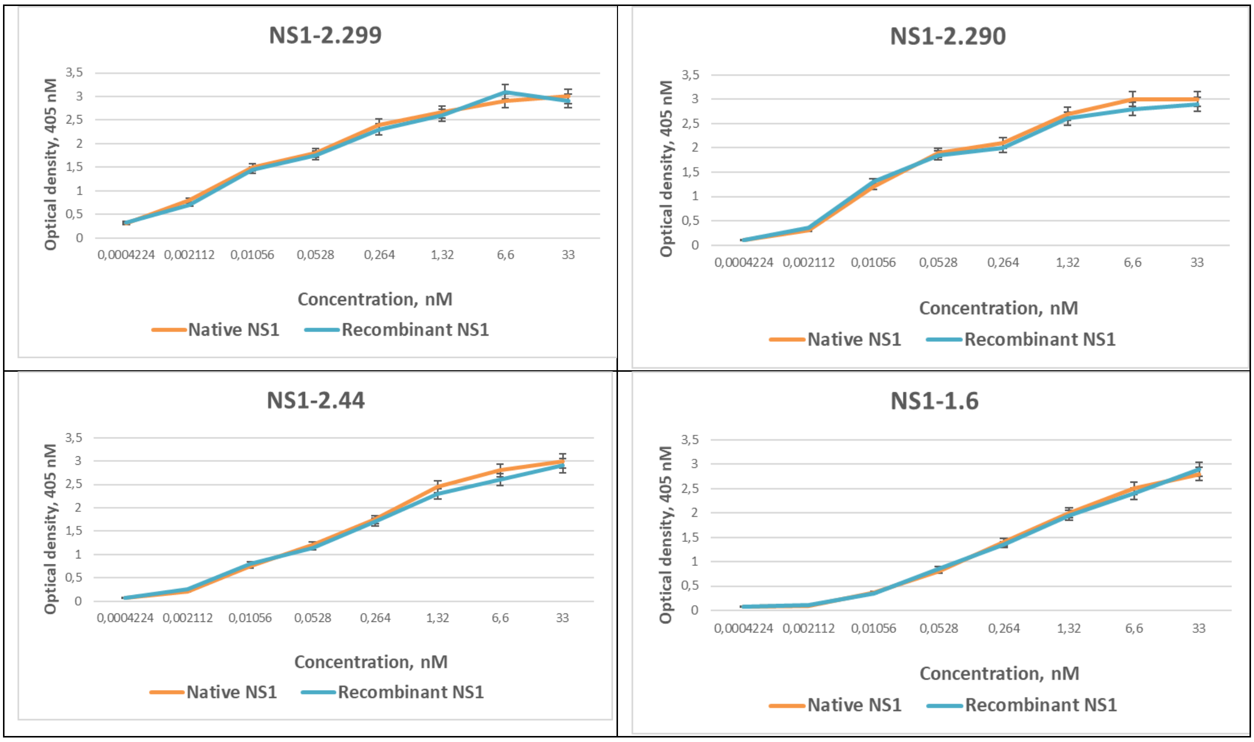

Figure S2. ELISA of serial dilutions of monoclonal antibodies NS1-1.6, NS1-2.299, NS1-2.290 and NS1-2.44 monoclonal antibodies specific to native TBEV NS1 to bind recombinant and native TBEV NS1 proteins.

Table S1. Top 20 genes according to the degree of change in mRNA amounts in TBEV NS1-treated HLMVEC cells compared to control samples.

№	Gene symbol	Function	K	NS1	logFC
1	CSF2	A cytokine that regulates the formation, differentiation and function of granulocytes and macrophages	1	593	9.2
2	SELE	Regulation of endothelial cell adhesion in response to cytokines	22	9363	8.7
3	VCAM1	Sialoglycoprotein expressed by cytokine-activated endotheliocytes. Regulates cell adhesion	19	2247	6.9
4	CXCL3	Secreted growth factor, chemoattractant for neutrophils	37	3844	6.7
5	C2CD4A	Participation in the development of acute inflammatory response, regulation of cell adhesion	7	437	6.0
6	CCL20	Through the receptor CCR6 attracts dendritic cells, T and B lymphocytes	9	480	5.7
7	TNFAIP6	A regulator of extracellular matrix during inflammation that binds to hyaluronan	0	53	5.7
8	LITAF	TNF induced by lipopolysaccharides	1	53	5.7
9	CX3CL1	A chemokine that regulates adhesion and migration on endothelium	116	6013	5.7
10	CXCL2	A chemokine expressed at sites of inflammation	70	3592	5.7
11	CXCL10	A chemokine that regulates monocyte function, migration of natural killer and T cells, and expression of adhesion molecules	0	37	5.2
12	NR4A3	Transcriptional activator	1	34	5.1
13	MAP3K8	Ser/Thr-kinase, activates MAPK and JNK pathways, triggers TNFα and IL-2 synthesis	11	363	5.0
14	NEURL3	E3 ubiquitin ligase, involved in lung development and innate immunity processes	2	64	5.0
15	ELOVL7	Fatty acid elongase, elongates unsaturated fatty acids C18:3 (n-3), C18:3 (n-6)-CoA, C20:4, C18:0, C18:1, C18:2, C16:0-CoA	1	28	4.8
16	ACKR3	Chemokine receptor CXCL11, CXCL12.	0	27	4.8
17	HAS3	Hyaluronan synthase	29	780	4.7
18	C1orf95	Integral protein	3	80	4.7
19	CSF3	IL-6 superfamily cytokine, regulates granulocyte formation, differentiation and function	167	3927	4.6
20	GPR37L1	G-protein coupled receptor	0	23	4.5

Table S2. Top 20 genes with significantly increased mRNA counts to over 500 in TBEV NS1-treated HLMVEC cells compared to control samples.

№	Gene symbol	Function	K	NS1	logFC
1	CSF2	A cytokine that regulates the formation, differentiation and function of granulocytes and macrophages	1	593	9.2
2	SELE	Regulation of endothelial cell adhesion in response to cytokines	22	9363	8.7

3	VCAM1	Sialoglycoprotein expressed by cytokine-activated endotheliocytes. Regulates cell adhesion	19	2247	6.9
4	CXCL3	Secreted growth factor, chemoattractant for neutrophils	37	3844	6.7
5	CX3CL1	A chemokine that regulates adhesion and migration on endothelium	116	6013	5.7
6	CXCL2	A chemokine expressed at sites of inflammation	70	3592	5.7
7	HAS3	Гиалуронан-синтаза	29	780	4.7
8	CSF3	IL-6 superfamily cytokine, regulates granulocyte formation, differentiation and function	167	3927	4.6
9	CXCL1	Growth factor acting through the CXCR2 receptor, a chemoattractant for neutrophils	1521	27332	4.2
10	CCL2	A cytokine that attracts monocytes and basophils	379	6399	4.1
11	RND1	A member of the Rho-GTPase family, it regulates the organization of the actin cytoskeleton in response to extracellular growth factors	86	1165	3.8
12	SOD2	Superoxide dismutase 2	2740	36222	3.7
13	C2CD4B	Regulation of inflammatory response, cell adhesion, vascular permeability	128	1554	3.6
14	TRAF1	TNF receptor-associated factor regulates NFκB and JNK activation	129	1537	3.6
15	BIRC3	E3 ubiquitin ligase that inhibits apoptosis by binding to TRAF1	259	3060	3.6
16	ICAM1	A surface glycoprotein expressed on endotheliocytes and immune system cells	2619	23541	3.2
17	TNFAIP2	Induced in response to TNFα	1948	16836	3.1
18	ZC3H12A	Endoribonuclease, regulates the inflammatory response by triggering degradation of translated cytokine-induced mRNAs associated with inflammation	140	1204	3.1
19	NFKBIZ	Regulates NFκB transfactor complexes	306	2631	3.1
20	CXCL8	IL-8, attracts neutrophils, basophils and T cells	4739	37383	3.0

Table S3. Gene-enriched metabolic and signaling pathways with increased expression levels (at least 4-fold) in TBEV NS1-treated HLMVEC cells compared to control samples

Enrichment FDR	nGenes	Pathway Genes	Fold Enrichment	Pathway
$1.2*10^{-31}$	18	112	108	TNF signaling pathway
$8.9*10^{-10}$	6	49	82	Malaria
$3.2*10^{-17}$	11	93	79	IL-17 signaling pathway
$2.0*10^{-13}$	9	92	66	Rheumatoid arthritis
$8.7*10^{-15}$	10	104	64	NF-kappa B signaling pathway
$1.6*10^{-7}$	5	57	59	Legionellosis
0.0001	3	37	54	African trypanosomiasis
$2.3*10^{-11}$	8	99	54	Viral protein interaction with cytokine and cytokine receptor
$4.1*10^{-7}$	5	70	48	Epithelial cell signaling in Helicobacter pylori infection

$2.3*10^{-6}$	5	100	34	AGE-RAGE signaling pathway in diabetic complications
$2.4*10^{-6}$	5	102	33	Amoebiasis
$9.2*10^{-11}$	9	191	32	Chemokine signaling pathway
$1.7*10^{-9}$	8	180	30	NOD-like receptor signaling pathway
$2.2*10^{-10}$	9	214	28	Lipid and atherosclerosis
$5.2*10^{-12}$	11	294	25	Cytokine-cytokine receptor interaction
$1.0*10^{-7}$	7	194	24	Kaposi sarcoma-associated herpesvirus infection
$1.1*10^{-5}$	5	141	24	Alcoholic liver disease
0.0002	4	138	19	Fluid shear stress and atherosclerosis
$4.7*10^{-5}$	5	192	17	Transcriptional misregulation in cancer
0.0001	5	232	14	Coronavirus disease
